# Machine learning-based prediction of mortality in pediatric trauma patients

**DOI:** 10.3389/fped.2025.1522845

**Published:** 2025-02-27

**Authors:** Alex Deleon, Anish Murala, Isabelle Decker, Karthik Rajasekaran, Alvaro Moreira

**Affiliations:** ^1^ Long School of Medicine, UT Health San Antonio, San Antonio, TX, United States; ^2^ Division of Neonatology, Department of Pediatrics, UT Health San Antonio, San Antonio, TX, United States; ^3^ Department of Otorhinolaryngology, University of Pennsylvania, Philadelphia, PA, United States

**Keywords:** machine learning, trauma, mortality, prediction, pediatrics

## Abstract

**Background:**

This study aimed to develop a predictive model for mortality outcomes among pediatric trauma patients using machine learning (ML) algorithms.

**Methods:**

We extracted data on a cohort of pediatric trauma patients (18 years and younger) from the National Trauma Data Bank (NTDB). The main aim was to identify clinical and physiologic variables that could serve as predictors for pediatric trauma mortality. Data was split into a development cohort (70%) to build four ML models and then tested in a validation cohort (30%). The area under the receiver operating characteristic curve (AUC) was used to assess each model's performance.

**Results:**

In 510,381 children, the gross mortality rate was 1.6% (*n* = 8,250). Most subjects were male (67%, *n* = 342,571) and white (62%, *n* = 315,178). The AUCs of the four models ranged from 92.7 to 97.7 with XGBoost demonstrating the highest AUC. XGBoost demonstrated the highest accuracy of 97.7%.

**Conclusion:**

Machine learning algorithms can be effectively utilized to build an accurate pediatric mortality prediction model that leverages variables easily obtained upon trauma admission.

## Introduction

Pediatric trauma is a significant global health challenge, contributing substantially to mortality and disability among children (1). The treatment of pediatric patients, who differ markedly from adults in their physiological, developmental, and psychological characteristics, requires a tailored approach. Despite advancements in pediatric trauma care, there remains a critical need for more refined risk stratification models to better predict patient outcomes and guide interventions. Current assessment measures often focus on immediate clinical parameters and outcomes; however, the incorporation of broader considerations, including post-discharge recovery, psychological support, and long-term rehabilitation, remains largely unexplored (2).

The complexities of pediatric trauma recovery challenge clinicians to identify ways to improve functional outcomes, one such method being the implementation of predictive models.

Machine learning (ML) has emerged as a powerful tool in healthcare, offering the ability to analyze vast datasets and make predictions with high accuracy (3). Its strength is in its ability to handle large amounts of data and describe non-linear relationships. To date, there has been some implementation of this technology to create predictive and risk assessment models in the setting of traumatic injuries (4). For instance, recent studies have demonstrated the potential of ML in predicting outcomes for pediatric traumatic brain injury (TBI) with moderate success, such as an Area Under the Receiver Operating Curve (AUC) of 0.78 (5). An AUC of 0.50 indicates that the model is no better at distinguishing diseased vs. non diseased individuals than chance, while an AUC of 1.0 indicates perfect discrimination. An AUC greater than 0.80 is considered clinically useful, respectively (6). Other models have utilized objective clinical data to create machine learning models that perform risk assessment in pediatric patients with blunt traumatic injuries (7–9). However, there is a notable gap in the literature regarding the development of high-performing ML models to predict mortality risk in children across a broad spectrum of traumatic injuries (10). This study aims to address this gap by developing an ML-based model that accurately predicts mortality risk in pediatric trauma patients, thereby enhancing triage and stratification processes in clinical settings.

## Methods

### Study design and setting

This retrospective cohort study utilized data from the National Trauma Data Bank (NTDB), the largest repository of trauma data globally, encompassing records from over 900 accredited trauma centers across the United States (11). The NTDB provides a comprehensive dataset ideal for developing predictive models due to its extensive range of clinical and demographic variables. We extracted data from the NTDB covering the period from 2007 to 2016, focusing exclusively on pediatric patients aged 18 years and younger. Data processing and analysis were conducted using the open-source R software. This study adheres to the Transparent Reporting of a Multivariable Prediction Model for Individual Prognosis or Diagnosis (TRIPOD) guidelines (12).

### Inclusion criteria

**Figure 1:**
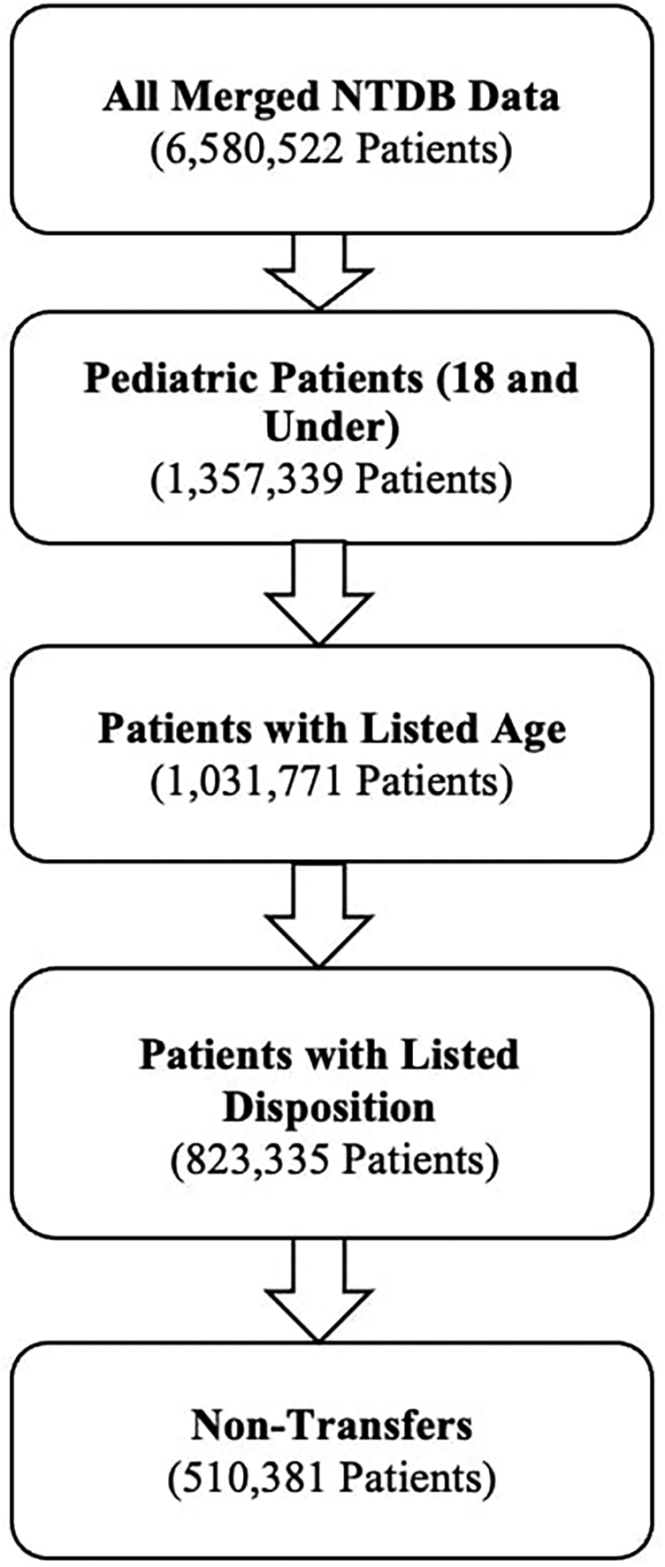
Flow diagram detailing the selection of NTDB patients.

The initial dataset comprised 6,580,522 patients treated for traumatic injuries between 2007 and 2016. Exclusion criteria were applied sequentially: 325,568 entries with missing age data were excluded, followed by the exclusion of 208,436 records lacking disposition information, and finally, 312,954 transfer patients were removed to focus on primary treatment outcomes. Our final dataset consisted of 510,381 incomplete patients. Figure 1 represents the process by which these patients were filtered and included in our final sample.

To address missing data in this cohort, multiple imputation was performed using R, with imputation rates ranging from 5%–10%. We initially applied a threshold of <20% missingness as the criterion for imputing data. Any variables with >20% missing values were excluded from the analysis to ensure data integrity and reduce the risk of bias from excessive imputation. We employed predictive mean matching and simple bootstrapping methods, implemented via the *Hmisc* package (v.4.6-0), to handle missing data for continuous and categorical variables, respectively. This approach was chosen due to its robustness in preserving variable distributions. Missing values were imputed iteratively (*n* = 10 times), and the results were assessed for consistency. To verify the imputation's robustness, we conducted sensitivity checks by comparing the percentages and distributions of imputed vs. original data, confirming that the distributions before and after imputation were nearly identical. This ensured that the imputation process did not introduce significant biases or distort the underlying data patterns.

### Outcomes and predictors

The primary outcome of this study was in-hospital mortality. The features used to make these predictions were based on data available at the time of emergency department arrival. These predictors included age, gender, race, mechanism of injury, type of injury (blunt vs. penetrating), intent of injury, location of injury, Injury Severity Score (ISS), systolic blood pressure, respiratory rate, temperature, and Glasgow Coma Score (GCS).

### Statistical analysis

Baseline characteristics between survivors and non-survivors were compared using the Wilcoxon rank-sum test for continuous variables, reported as medians with interquartile ranges (IQRs), and the Chi-square test for categorical variables, reported as frequencies and percentages. Statistical significance was defined as a *p*-value < 0.05. A 10-fold cross-validation scheme was employed, repeated five times for enhanced generalization and to reduce variability in performance metrics. The trainControl function from the *caret* package was configured with twoClassSummary as the summary function to compute key evaluation metrics (e.g., sensitivity, specificity, and AUC) for binary classification tasks.

The dataset was randomly partitioned into a training set (70%) and a testing set (30%) to evaluate model performance. Four distinct machine learning models were used to analyze the predictors outlined earlier in this paper: multivariate adaptive regression spline (MARS), partial least squares (PLS), deep neural networks (Nnet), and eXtreme gradient boosting (XGBoost).

• **MARS** is a non-parametric regression technique that models complex relationships by combining simple linear splines (13). MARS models are well suited to uncover complex, non-linear patterns among predictor variables without requiring transformation of data.
• **PLS** is a dimensionality reduction technique that models the relationship between independent variables and outcomes in high-dimensional datasets (14). PLS models are effective at reducing the dimensionality of datasets with collinear predictors (e.g., vital signs, GCS, mechanism of injury, etc), a common finding in trauma datasets.
• **Nnet** is an artificial neural network algorithm that mimics brain neuron connections to identify patterns and make predictions (15). Its ability to model relationships between input variables and outcomes makes it especially relevant for prediction of outcomes in trauma patients
• **XGBoost** is an ensemble method using gradient boosting of decision trees, known for its high performance and efficiency in handling large datasets (16). These models provide scalability and high predictive accuracy in the analysis of large amounts of data.

These models represent a diverse array of methodologies and perspectives that collectively address the multifaceted nature of trauma outcome prediction, ensuring a comprehensive analysis that leverages the strengths of each approach. Furthermore, each of these models is well suited for handling large datasets. The area under curve (AUC) was used to measure the effectiveness of the models, as it maps specificity (*x*) vs. sensitivity (*y*) to distinguish between two given parameters. Youden's index was utilized to determine an optimal threshold value to evaluate the sensitivity and specificity (17).

## Results

### Characteristics of study population

Table 1 demonstrates the demographics of our patients. The study included a total of 510,381 pediatric patients who sustained traumatic injuries between 2007 and 2016. The median age of the patients was 13 years (IQR: 6–16). The majority of the cohort were male (67%, *n* = 342,571) and White (62%, *n* = 315,578). The median ISS was 5 (IQR: 4–10), with significant differences observed between survivors and non-survivors. Specifically, survivors had a median ISS of 5 (IQR: 4–9), whereas non-survivors had a significantly higher median ISS of 29 (IQR: 25–41; *p* < 0.001).

**Table 1:** Demographics.

Characteristic	*N*	Overall, *N* = 510,381^a^	Survived, *N* = 502,131^a^	Died, *N* = 8,250^a^	*p*-value^b^
Age	510,381	13.0 (6.0, 16.0)	13.0 (6.0, 16.0)	16.0 (11.0, 17.0)	<0.001
Gender	510,381				<0.001
Female		167,810 (33%)	165,503 (33%)	2,307 (28%)	
Male		342,571 (67%)	336,628 (67%)	5,943 (72%)	
Race	510,381				<0.001
White		315,178 (62%)	310,850 (62%)	4,328 (52%)	
Asian		10,037 (2.0%)	9,891 (2.0%)	146 (1.8%)	
American Indian		3,616 (0.7%)	3,573 (0.7%)	43 (0.5%)	
Black or African American		106,708 (21%)	104,335 (21%)	2,373 (29%)	
Native Hawaiian or Other Pacific Islander		1,410 (0.3%)	1,391 (0.3%)	19 (0.2%)	
Other Race		73,432 (14%)	72,091 (14%)	1,341 (16%)	
ISS	510,381	5.0 (4.0, 10.0)	5.0 (4.0, 9.0)	29 (25, 41)	<0.001

^a^ Details the format of the column as listed below it. “*n* (%)” is the format used in the mechanism of injury column and “Median (IQR)” is the format used for the variables below.

^b^ References to the tests used to generate these *p*-values.

Patient characteristics and mechanisms of injury are described in Table 2. We identified 8,250 (1.6%) patients who died during their hospitalization. Motor vehicle collisions were a major cause of mortality accounting for 60.9% (*n* = 5,024) of the deaths. Injuries sustained by firearms were particularly deadly, accounting for 3.8% (*n* = 19,394) of the total cohort's injuries but comprising 20% (*n* = 1,620) of all deaths. SBP, respiratory rate, temperature, and GCS were all significant predictors of mortality.

**Table 2:** Mechanism of injury and patient characteristics.

Characteristic	*N*	Overall, *N* = 510,381^a^	Survived, *N* = 502,131^a^	Died, *N* = 8,250^a^	*p*-^b^
Mechanism of Injury	510,381				<0.001
Cut/pierce		18,792 (3.7%)	18,622 (3.7%)	170 (2.1%)	
Fall		118,932 (23%)	118,617 (24%)	315 (3.8%)	
Firearm		19,464 (3.8%)	17,844 (3.6%)	1,620 (20%)	
MVT Motorcyclist		8,057 (1.6%)	7,897 (1.6%)	160 (1.9%)	
MVT Occupant		190,033 (37%)	186,395 (37%)	3,638 (44%)	
MVT Other		1,749 (0.3%)	1,698 (0.3%)	51 (0.6%)	
MVT Pedal cyclist		9,846 (1.9%)	9,645 (1.9%)	201 (2.4%)	
MVT Pedestrian		30,361 (5.9%)	29,471 (5.9%)	890 (11%)	
MVT Unspecified		1,313 (0.3%)	1,230 (0.2%)	83 (1.0%)	
Pedal cyclist, other		17,912 (3.5%)	17,875 (3.6%)	37 (0.4%)	
Pedestrian, other		2,285 (0.4%)	2,219 (0.4%)	66 (0.8%)	
Struck by, against		35,599 (7.0%)	35,389 (7.0%)	210 (2.5%)	
Transport, other		25,133 (4.9%)	24,861 (5.0%)	272 (3.3%)	
SBP	510,381	124 (112, 137)	124 (113, 137)	108 (72, 136)	<0.001
Pulse	510,381	99 (84, 115)	99 (84, 115)	103 (67, 133)	0.016
RR	510,381	20.0 (18.0, 24.0)	20.0 (18.0, 24.0)	16.0 (0.0, 20.0)	<0.001
Temperature	510,381	36.7 (36.2, 37.0)	36.7 (36.2, 37.0)	36.0 (35.0, 36.7)	<0.001
GCS	510,381	15.0 (15.0, 15.0)	15.0 (15.0, 15.0)	3.0 (3.0, 3.0)	<0.001

*n* (%); Median (IQR).

Pearson's Chi-squared test; Wilcoxon rank sum test.

^a^ Details the format of the column as listed below it. “*n* (%)” is the format used in the mechanism of injury column and “Median (IQR)” is the format used for the variables below.

^b^ References to the tests used to generate these *p*-values.

### ML prediction of death outcome

**Figure 2:**
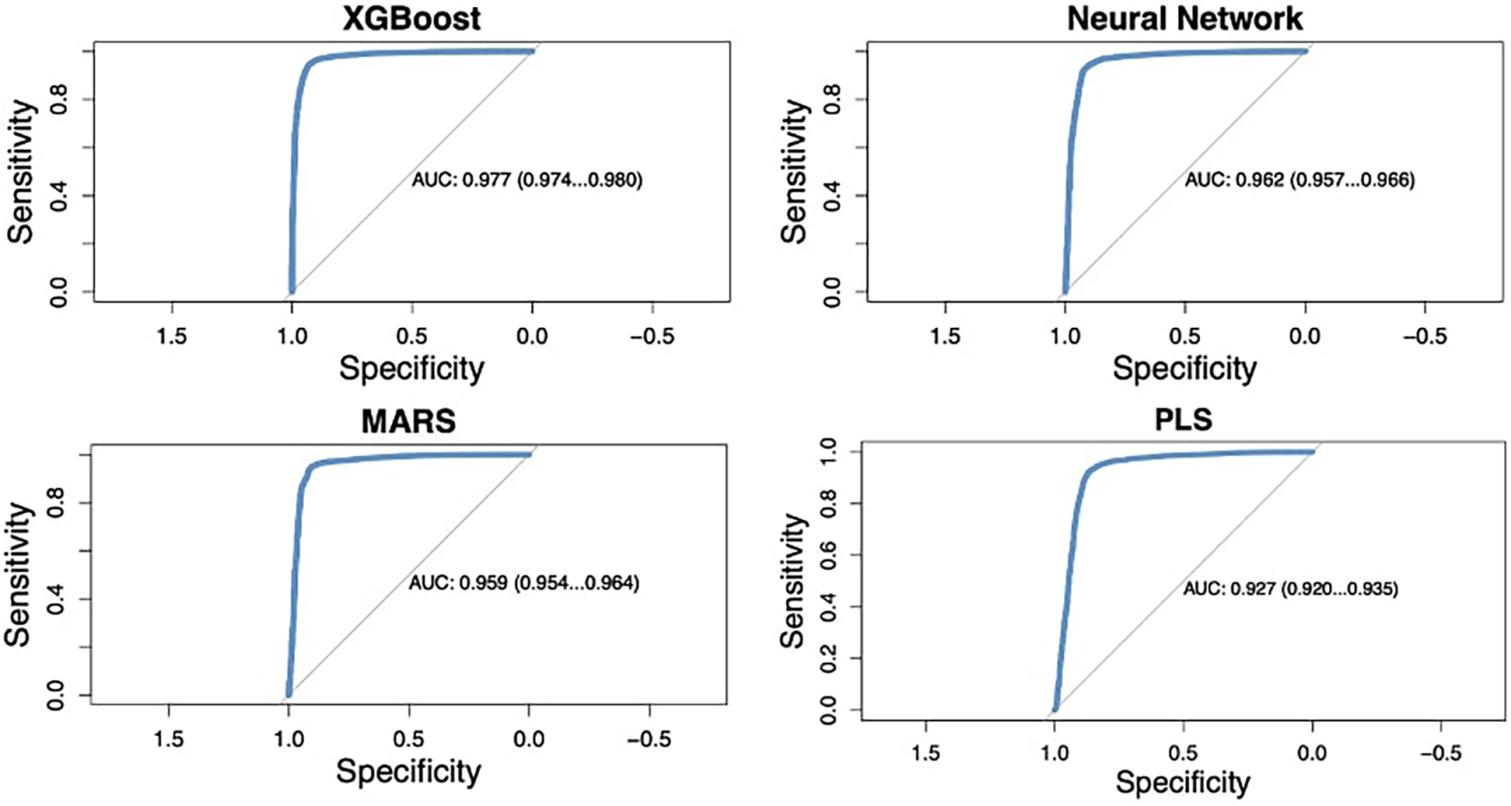
Area under the curve (AUC) by model.

The four machine learning models evaluated in this study demonstrated high predictive accuracy. The AUC for the models ranged from 92.7% to 97.7% (Figure 2). XGBoost outperformed the other models, achieving an AUC of 97.7% (95% CI: 97.4%-98.0%), with a sensitivity of 94.7% and a specificity of 92.9% at the optimal threshold determined by Youden's index. The deep neural network (Nnet) model also performed well, with an AUC of 96.2% (95% CI: 95.7%–96.6%), a sensitivity of 93.0%, and a specificity of 91.6%. The performance metrics for each model are summarized in Table 3.

**Table 3:** Predictors of performance.

Model	AUC (95% CI)	Youden's Index	
		Sensitivity	Specificity
XGBoost	97.7 (97.4–98.0)	94.6%	92.9%
Neural Network	96.2 (95.7–96.6)	93.0%	91.6%
MARS	95.9 (95.4–96.4)	94.7%	90.8%
PLS	92.7 (92.0–93.5)	92.2%	86.9%

### Variable importance

**Figure 3:**
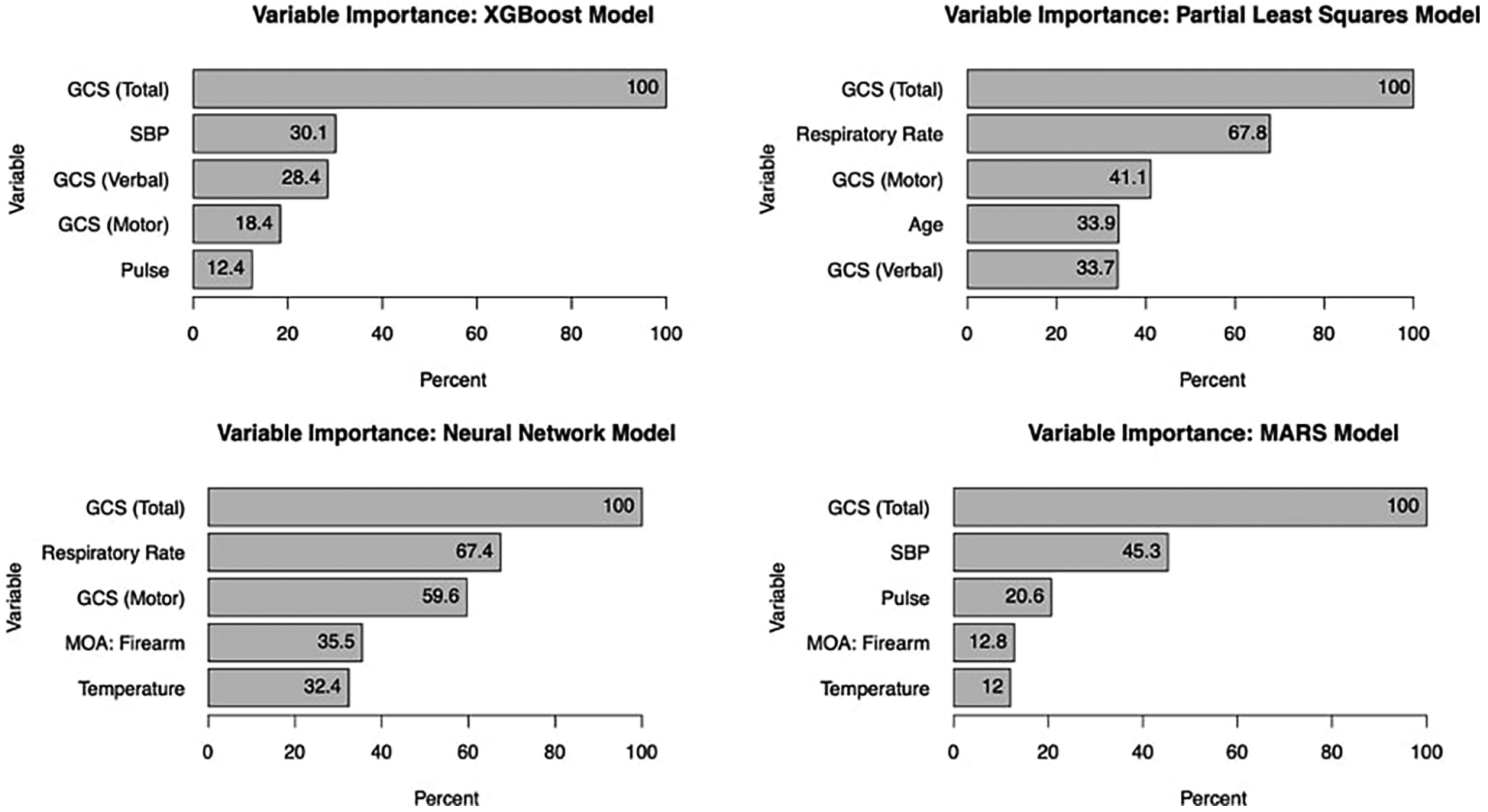
Variable importance by model.

Glasgow Coma Score (GCS) consistently emerged as the most significant predictor of mortality across all models, with an importance score of 100%. For the XGBoost model, the top five predictors were GCS, systolic blood pressure, respiratory rate, temperature, and mechanism of injury (with firearm-related injuries being particularly influential). The MARS model identified GCS, systolic blood pressure, pulse, temperature, and mechanism of injury as the most informative variables, highlighting the complex interplay between these factors in predicting in-hospital mortality (see Figure 3).

## Discussion

In this study, we successfully developed and validated machine learning models to predict mortality in pediatric trauma patients using variables readily available at the time of emergency department (ED) admission. Among the four models tested, XGBoost demonstrated superior performance with an AUC of 97.7%, underscoring its potential utility in clinical settings for real-time risk stratification. With an importance of nearly 100% in each of our four models, GCS was identified as a crucial factor in understanding and predicting outcomes. GCS is a widely used clinical tool for assessing a patient's level of consciousness following a traumatic injury. Its proven effectiveness in predicting mortality, combined with its ease of use and simplicity, makes it well suited for use in machine learning model prediction (18–20).

This study revealed significant sociodemographic discrepancies in patient outcomes. Our results showed a lower mortality rate for White patients (1.37%) when compared to Black patients (2.22%). In a similar review, Hakmeh et al. found that Black pediatric trauma patients experienced higher mortality rates (7%) than White pediatric trauma patients (4%). This discrepancy was further exacerbated by the insurance status of the patients (21). Uninsured patients had a higher mortality rate than those with health insurance, which may be linked to the fact that Black patients are more likely to be uninsured than White patients (11% of Black patients were uninsured compared to 7% of White patients in 2021) (22). Black patients may face unique barriers to healthcare, such as fewer trauma centers in their geographic areas or limited resources at the facilities they are able to access (23). This population also suffers more penetrating and nonaccidental trauma (24). In the setting of pediatric trauma, the observation of a disparity in outcomes for White vs. Black patients is consistent with the findings of similar models that have analyzed NTDB data. This finding further validates the efficacy of our machine learning model as a predictor of patient outcomes. These findings call for providers to deliver culturally competent care to trauma patients, regardless of racial or cultural differences.

The data used in this study was from the NTDB and included 70,781 patients with ISS > 8, which is a moderate-risk group for mortality. Firearms were found to be the deadliest mechanism of injury, accounting for 20% of deaths despite comprising only 3.8% of total cases. This finding was corroborated by Lee et al., who reported that children and youth in the United States succumb to firearm injuries at a rate of more than 10 times that of all other developed countries (as designated by the Organization for Economic Cooperation and Development) combined (25). Furthermore, this study concluded that firearm homicides and suicides in US youth have increased by 14% and 39% respectively, which emphasizes the need for optimization of treatment strategies.

Although motor vehicle collisions are a major cause of injury in the pediatric age group, this could be attributed to the sheer number of cases rather than the lethality of the mechanism itself. A total of 241,359 motor vehicle related cases were observed in this study compared with 19,464 firearm related cases. A study by Theodorou et al. found that firearm and motor vehicle collisions are two of the most common causes of injury among children and adolescents, yet firearm violence has a case fatality rate 50 times higher than injuries caused by motor vehicle collisions (26). This further emphasizes the point that although injuries caused by both firearms and motor vehicle collisions are the cause of a majority of the deaths in the pediatric age group, firearm injuries are inherently the most lethal mechanism of injury: based on our results, a pediatric patient with a firearm-related injury is significantly more likely to die than a patient sustaining injuries from a motor vehicle collision.

The variables used in our models have been previously used to construct robust and validated models. In a similar study analyzing NTDB data to predict trauma mortality, Tsiklidis et al. used systolic blood pressure, heart rate, respiratory rate, temperature, oxygen saturation, gender, age, and GCS to build a gradient boosting model with an AUC of 0.924 (27). In comparison to this study, our study demonstrates several advantages. While the Tsiklidis study also used NTDB data, their data was confined to one single year (2016) whereas we looked at data across multiple years. Another well-known study implemented machine learning models to predict clinical outcomes in the emergency room setting (28). Using a dataset of 135,470 adult patients, this study created four highly accurate machine learning models that outperformed a reference logistic regression model in predicting hospitalization and critical care needs, as well as providing a net benefit in accurately triaging patients. Although our primary outcomes are different (admission outcome vs. mortality), their most important variables were similar to ours. However, our study demonstrates some advantages. Our sample size of over 500,000 patients vs. their 135,740 patients gives our models stronger credibility. Additionally, the range of our AUCs (92.7–97.7) was comparably higher than this particular study's (81.0–86.0). Another relevant study has used machine learning algorithms to predict mortality in pediatric warzone patients with a performance of up to 97.5% (29). While these models performed similarly to ours, they were trained using a small group of 2,007 pediatric trauma patients sustaining injuries in an austere setting. Lastly, we identified one large Korean study that created artificial intelligence models to predict mortality in emergency department patients of all ages (30). While these models performed exceptionally well with an AUC as high as 99.7%, the predictions relied heavily on the Korean Triage and Acuity Scale (KTAS) and did not incorporate GCS. To the authors' knowledge, our study represents the first machine learning model created to predict mortality in pediatric trauma patients sustaining a broad range of injuries in the United States.

The strengths of our study include a large sample size from a large national data bank, while adhering to all 22 checklist items of the TRIPOD guidelines (12). Our analysis included data from entries across a span of 10 years, providing us with the opportunity to analyze a highly diverse group of patients. Additionally, our model was successfully internally validated and demonstrated higher accuracy in predicting mortality, with an XGBoost AUC of 0.977, when compared to other published models, such as the model by Tsiklidis et al. with an AUC of 0.924 or the Raita model with an AUC of 0.81–0.86. Our model uses variables that are readily available at the time of ED admission, making the deployment of this model for clinical use a realistic possibility. The advantage of our model is not only in mortality prediction, but also in its ability to identify variables that could serve as early warning signs in trauma patients. Early identification of such warning signs may not only assist physicians with patient assessment and triage, but also may aid in efforts to improve the efficiency of trauma systems (31). The simplicity of measuring our variables in the hospital setting makes our models highly efficient and transparent methods of predicting mortality. In a fast-paced trauma setting, such a model can be utilized by providers to identify key factors contributing to the instability or imminent deterioration of a trauma patient.

There are a few limitations to our study. Our models were only validated internally, not externally. Although internal validation is a relatively accurate method of validating a machine learning model, external validation further highlights the real-world applications of the model. Additionally, this study included retrospective data. Although we feel that this dataset is an accurate representation of current trauma data, a prospective study would provide a more complete sample set and possibly yield a higher performing predictive model. While we attempted to isolate some of the most crucial variables in determining clinical outcomes, it is possible that data points not included in the NTDB could be significant predictors of mortality, such as co-morbidities. We anticipate that future studies analyzing additional factors, such as economic and insurance status, may also shed light on this issue.

Overall, this study was able to develop a highly accurate prediction model for mortality in pediatric trauma patients using easily accessible variables upon ED admission. Identifying key predictors of mortality can help providers in the prioritization and structure of treatment in the acute trauma setting. The application of these models may lead to the development of quality improvement measures that address the observed disparities in the delivery of treatments. Our model proved to be more accurate than other high-performing models, providing a valuable tool for medical professionals to use in their decision-making processes. In the future, it is our hope that such a machine learning model may be converted to a web-based app that can accept clinical variables and predict mortality in real time. This may provide investigators with the unique opportunity to conduct prospective studies using a machine learning model. Additional investigations are also warranted to identify the performance of a machine learning model as patient treatment progresses through time.

## Data Availability

The data analyzed in this study is subject to the following licenses/restrictions: The NTDB dataset is available for purchase. Requests to access these datasets should be directed to https://www.facs.org/quality-programs/trauma/quality/national-trauma-data-bank/about-ntdb/.
