# Supplementary material for: Machine learning-based prediction of mortality in pediatric trauma patients
Source: Front Pediatr. 2025 Feb 27;13:1522845. doi: 10.3389/fped.2025.1522845 (PMC11905922; doi:10.3389/fped.2025.1522845)
Supplement: Supplementary file 1 [file Table1.docx]

1. Age
2. GCS
3. Gender
4. Injury Type
5. Injury Intent
6. Location of Injury Occurrence
7. Mechanism of Injury
8. Pulse
9. Race
10. Respiratory Rate
11. Systolic Blood Pressure
12. Temperature

Supplemental Figure 1: Variables Included as Predictors

Supplemental Figure 2: Aggregated AUCs for All Models

Supplemental Figure 3: Lift Curve

Supplemental Figure 4: Lift Curve 2

Supplemental Figure 6: Calibration Curve 1

Supplemental Figure 7: Calibration Curve 2
